# Openness and Communication Effects on Relationship Satisfaction in Women Experiencing Infertility or Miscarriage: A Dyadic Approach

**DOI:** 10.3390/ijerph17165721

**Published:** 2020-08-07

**Authors:** Ewa Kiełek-Rataj, Anna Wendołowska, Alicja Kalus, Dorota Czyżowska

**Affiliations:** 1 Institute of Psychology, University of Opole, 45-052 Opole, Poland; erataj@uni.opole.pl; 2 Institute of Psychology, Jagiellonian University, 30-060 Krakow, Poland; anna@wendolowska.com (A.W.); d.czyzowska@uj.edu.pl (D.C.)

**Keywords:** infertility, miscarriage, communication, openness, relationship satisfaction, actor–partner interdependence model

## Abstract

Openness and communication between partners are key elements of dyadic coping with stress. Our main research question is: what is the impact of these factors on relational satisfaction in spouses struggling with infertility or miscarriage? In the current study, by applying the actor–partner interdependence model to 90 heterosexual couples (*N* = 180), we examined the link between the spouses’ openness (the Giessen Test), communication (Flexibility and Cohesion Evaluation Scales) and relationship satisfaction (the Marriage Success Scale). Controlling for relevant covariates (communication, own openness and type of stress experienced by the spouses: infertility or miscarriage), a dyadic analysis revealed significant actor (−0.24; *p* < 0.001) and partner effects (−0.20; *p* < 0.001). We conclude that the relationship between the perception of the partner’s openness and the relationship satisfaction in women is strong, in the context of the analyzed potential confounding variables. We also observe that the relationship satisfaction in women from the group of infertile spouses is 6.06 points lower compared to women from the group of marriages after miscarriage (*p* = 0.034).

## 1. Introduction

The emergence of stress in spouses’ lives significantly reduces their satisfaction with the relationship [1]. Openness in a relationship, understood as the ability to reveal one’s feelings, thoughts, needs and fears, is associated with a higher satisfaction with the relationship [2,3,4], and its lack leads to conflicts and a breakdown of the relationship [5]. Openness is one of the key elements of high-quality communication between partners [4], which in turn allows them to effectively coping with stress [6,7]. Open interpersonal communication is one of the manifestations of openness understood as a broader concept referring to the general attitude of partners towards each other and the world [8]. Although openness determines the quality of communication between spouses [4], sharing difficult emotions may result in a decrease in satisfaction with the relationship [4,9]. Effective communication under stress—which is based not only on openness, but also on mindfulness [10], commitment [11], an accurate recognition of the partner’s feelings [12] and the ability to accept others’ points of view [13]—can reduce the negative effects of interpersonal conflicts in the face of stress experienced by couples [14] and results in a higher satisfaction with the relationship [15,16,17,18]. What is the role of communication and openness in relationships struggling with traumatic experiences?

Undoubtedly, such relationships include infertile couples and those who have gone through a miscarriage. Infertility is medically defined as the inability to conceive after a year or more of regular, unprotected sexual intercourse [19]. It is estimated that 8–12% of couples around the world experience difficulty conceiving a child [20]. Although the extent of infertility varies considerably among countries, infertility has been recognized as a worldwide public health issue by the World Health Organization (WHO) and has the potential to threaten the stability of individuals, relationships and communities [21,22]. About 10–15% of pregnancies end in miscarriage [23,24,25,26,27], which is defined by the World Health Organization [28] as “the accidental loss of a fetus before a full term pregnancy, resulting in the death of the fetus”. The frequency of this phenomenon increases as the age of the mother increases: 30–50% of women experience anxiety symptoms after miscarriage, and 10–15% experience depressive symptoms [29] as a result of grieving after losing a child.

The experience of both the studied groups is loss. For spouses who lost their child before delivery, this means the loss of a child who already existed. For spouses struggling with diagnosed infertility, it is the loss of a child who was not conceived despite the efforts of the couple. In both cases, these are traumatic events that concern all affected women [29,30,31]. The studies conducted so far on couples facing fertility problems have mostly focused on stress and its consequences for relationship functioning [32] and the quality of life of both partners [33,34]. The issue of miscarriage has been less frequently studied, but attempts have been made to explore the impact of miscarriage on communication [35,36,37] and on interpersonal and sexual relations between partners [35,38,39,40].

The current study was intended to consider relationship satisfaction with reference to the openness of the partners and quality of their communication. In a marital relationship, two people cannot be considered totally independent of each other [41]. The examination of interpersonal processes requires that the spouses’ data be considered as interdependent rather than independent [42], which means that a characteristic or behavior of one person affects his or her partner’s outcomes [43]. The actor–partner interdependence model approach uses the dyad, not the individual, as the sampling unit and provides separate but simultaneous estimates of actor and partner effects [43]. The actor effect assesses the degree to which one’s outcome is influenced by one’s own characteristics, whereas the partner effect assesses the degree to which a person’s outcome is influenced by the partner’s characteristics.

### 1.1. Infertility and Relationship Satisfaction

Infertility is considered one of the most stressful life events [44,45] and personal tragedies [46]. The inability to have a child affects the emotional and psychological state of both partners and is not without impact on their mutual relations [47] and their relationship satisfaction (e.g., [48,49]). Research on infertile couples demonstrates the interdependence between marital satisfaction, sexual satisfaction, and quality of life [50,51,52,53].

It often happens that the desire to have a child dominates over other wishes and ambitions in life [54]. For many couples, and especially for women, fertility problems become a source of great stress because they are perceived as a crisis or failure in life [55]. While this is difficult for both partners, infertility, its diagnosis and treatment are considered particularly stressful by women. They also suffer a more pronounced deterioration of well-being, which negatively affects their female identity [56,57,58]. It should be noted, however, that although men seem to be able to cope better with the problem, infertility greatly reduces their sense of masculinity [59,60,61]. Infertility and related stress affect satisfaction with the relationship and how its quality is assessed. Research shows that women diagnosed with infertility report lower relationship satisfaction than fertile women [56,60,62,63]. The relationship between infertility and satisfaction in women is mediated by a number of factors, such as representations about the importance of parenthood and the perceived impact of infertility on various life domains for which satisfaction and resilience are the moderators of this relationship [64]. The greater a woman’s relationship satisfaction and resilience, the lower the impact of the importance attributed to parenthood on her life satisfaction.

Research aimed at establishing the relationship between the stress experienced by infertile spouses and the marital satisfaction of partners shows that perceived stress affects the feeling of satisfaction with the marriage [65]. The stress perceived by women has a negative impact on their partner’s marital satisfaction. Although the stress perceived by the male partner seemed to have no influence on the woman’s satisfaction, wives whose husbands reported higher levels of stress were more likely to feel less satisfied with the relationship. Stress perceived by both partners affects the relationship satisfaction of infertile spouses. The stress of each partner also affects their level of sexual satisfaction. At the same time, the greater the infertility-related sexual concerns in men and women, the lower the level of sexual satisfaction for both partners. A meta-study on infertile couples [66] identified the importance of infertility for mental well-being, marital relationships, sexual relationships and quality of life. It has been established that infertility has a negative impact on the mental well-being and sexual relations of couples. The results regarding the influence of infertility on marital relationships and quality of life were inconclusive. Some studies report that spouses struggling with infertility also recognize the positive aspects of this experience, namely an increased sense of intimacy and proximity [57]. While studies of infertile women treated with assisted reproductive technology have shown that the positive effects of treatment are associated with lower interpersonal intimacy and reluctance to reveal oneself in a romantic relationship [67].

### 1.2. Impact of Miscarriage on Communication and Marital Satisfaction

Maternity is an important period in a woman’s life. Many biological, psychological, and social changes in women begin as early as conception: their self-image changes, as does their concept of their psychological, physical and social self [68]. The woman adapts her lifestyle to the requirements of her body [69]. The gestation period is when the bond between mother and child is being formed [70,71]. Therefore, losing a child during the prenatal period interrupts the natural development of this emotional bond [72,73]. This event is of great importance and at the same time it is very stressful. How partners cope with this, how they experience grief and what emotions arise at this time all affects the mutual relationship between partners [74].

The issue of miscarriage is rarely discussed in the literature. Few authors try to determine how the prenatal loss of a child affects interpersonal and sexual relations between partners [35,38,39,40] or how it disrupts good communication [35,36,37]. Losing a child during the prenatal period may have repercussions on the spouses’ interpersonal relations and the quality of their sexual life [39,40]. It is noteworthy that women who had experienced miscarriages and were in close, caring relationships with open communication patterns coped better with the emotional consequences of the miscarriage, therefore the loss was not so overwhelming for them [40].

The loss of a child in the prenatal period and the associated sadness can lead to disorders and communication difficulties between spouses [35,36,37]. Problems most often result from differences in the way women and men communicate. Often, parents who have suffered a miscarriage decide not to talk about this experience, instead trying to guess what the other person needs [38]. Men usually do not express their feelings and emotions openly [39]. Women who need comfort and closeness can interpret their partner’s attitude as indifference to loss, a disregard for her pain, and lack of affection for the lost child [35,38]. Without proper communication, partners unnecessarily misunderstand each other, and the marital subsystem is destabilized [35].

Studies show gender differences in coping strategies with miscarriage: most women want to talk about loss, while men usually avoid this topic [39] for fear of saying something wrong [36,40]. Qualitative research based on interviews conducted with couples who have suffered a miscarriage indicates that the partners had different expectations as to how they would experience mourning [36]. Divergent expectations caused marital tensions and limit the support partners could provide to each other. In addition, misunderstandings and discrepancies in defining the sense of loss and the “proper” way of experiencing mourning caused the spouses to move away from each other emotionally [36]. As researchers indicate, open communication and honest conversations can help spouses survive the difficult period of the prenatal loss of a child [35,40,75].

Research highlights the role of mutual support in the miscarriage experience [35,38,72,76,77,78]. Above all, both spouses need safety, relief and support from their partner [35]. When experiencing the pain associated with the loss of a child, each spouse should also be a source of support for the partner, which can be a double burden [76,77,78]. The experience of miscarriage causes tension in the marriage subsystem and disturbs its balance [35], but the effect varies between marriages. Through this experience, some couples find that their relationship is able to survive such difficult times; for others, this experience may destabilize mutual relationships. Extensive studies conducted on a group of 3707 women show that the risk of relationship breakdown is much higher in couples who have experienced prenatal loss than in relationships that have not suffered such an experience [79]. The authors point out that losing a child in the prenatal period can be a source of additional stress in a relationship, and although most couples are able to cope with difficult situations, some may not be able to survive this challenge.

### 1.3. Openness and Its Significance for Relationship Satisfaction

Communication between partners is an important element of coping with stress. If it is open and clear, it opens up the opportunity to take effective action [6]. Communication based on adopting one’s partner’s perspective and expressing emotions safely can condition relationship satisfaction [80]. Communicating one’s feelings and concerns openly encourages relational satisfaction [68]. Research also shows that open communication about day-to-day positive events is linked to individual and relational well-being [3].

Not only is openness a key feature in a romantic relationship (e.g., [80,81,82]), but it also appears to be more important than expressing emotions or interacting on a regular basis [80]. It is at the same time one of the most desirable characteristics in a partner. Openness proves to be an important trait in deciding the quality of communication between partners [4], but it is also a major aspect of relationships in general [82]. It accounts for one’s subjective evaluation of the relationship [4] and is a key element of dyadic coping [3]. A lack of openness and implicit communication both increase the number of conflicts and, as a consequence, the risk of the relationship failing [5].

Research indicates that the direct communication of positive events contributes to relationship satisfaction in women and men alike [3]. However, communicating stressful experiences is different because it carries the risk of temporarily reducing relationship satisfaction [4,9], because it may threaten the sense of agency and efficacy of the person experiencing stress. On the other hand, explicit communication in a stressful situation increases the likelihood of the other person responding adequately as a result of getting the message right and showing support, understanding, and kindness towards the supported partner [3]. Findings show that partners who openly share their stress are more satisfied with their relationship [4], and the way the stressed partner chooses to communicate has an impact on the other partner’s dyadic coping [7].

Assessing a partner’s behavior as well as perceiving their emotions, intentions, and views is sometimes considered to be the most important predictor of relationship functioning [83]. In romantic relationships, individuals influence each other by giving meaning to their behaviors, which consequently forms their opinion on the quality of that relationship. The theory of interdependence in close relationships [84,85] relates to how partners interact with one another. It assumes that one partner’s perception and behavior are not independent of the other partner; quite the opposite, they are linked and both partners mutually affect each other’s actions and reactions [86,87].

When we examine relationship satisfaction, it should be noted that it depends on how one perceives one’s partner. Expectations of both oneself and the partner, and especially the perception of these expectations, are important factors in predicting the quality of relationship functioning. Research in this area indicates that when a partner is viewed in a more positive way, the relationship satisfaction is higher [5]. Seeing one’s partner positively—as capable of meeting one’s needs and seeking closeness in difficult times—can foster a sense of security in the relationship, thereby increasing the quality of its functioning [88]. Other studies have shown that, for the evaluation of relationship satisfaction, it is important to see prosocial motives in the partner’s behaviors and notice their openness to understanding the other person’s needs [84,89].

Studies on the perception of one’s own and partners’ interpersonal problems have indicated that the perception of a partner’s problems is much more important for relationship satisfaction than the perception of one’s own problems. A positive perception of problems is sometimes referred to as “idealization” in the literature, i.e., spouses who perceive their partners as causing fewer interpersonal problems are more satisfied with their relationship [90].

There can be no doubt that infertility or the loss of a child due to miscarriage are sources of stress and a heavy burden for both spouses, therefore they represent a threat to the relationship. It follows from the above that coping with stressful situations, and consequently relationship satisfaction, is dependent on the ways they communicate and the degree to which partners are ready to share and be open about their feelings and emotions. Partner perception and communication in the relationship are also important. In our study, the following questions were asked: How satisfied with their relationships are women who are infertile or have had a miscarriage? How is this related to the willingness of partners (understood as the ability to show love and intimacy) to be available to the partner and the desire to be open to them? What is the role of quality communication between partners? What is the relationship satisfaction in women with infertility or after a miscarriage? How is this related to the openness of the partners? What is the role of quality communication by both partners? By openness we mean the person’s ability to show love and intimacy, to be available to the partner and the desire to be open to them.

## 2. The Purpose of the Study

**Figure 1:**
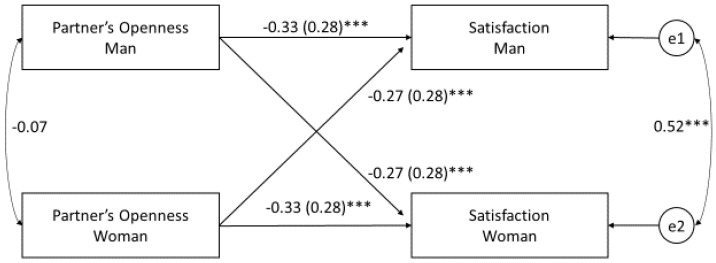
Perception of partner’s openness (opposite pole of reservedness) and relationship satisfaction. Base model. The rectangles represent the independent and dependent variables; the two circles present the latent error terms; the arrows describe the actor and partner effects. The curved double-headed arrows on the left represent the covariances between the independent variables; the curved double-headed arrow on the right represents the correlation between the two error terms (e1, e2: residual errors on satisfaction for males and females, respectively). * *p* < 0.05; ** *p* < 0.01; *** *p* < 0.001; standardized coefficients (*β*) are reported with standard error in parentheses.

The aim of the study was to determine the relationship between spouses’ openness and relationship satisfaction in women. We decided to examine two groups of spouses, each of which had faced a difficulty that could potentially influence their relationship satisfaction. One group comprised spouses who had experienced the loss of a child in the prenatal period; the other comprised spouses with diagnosed infertility. We expected significant correlations between partner’s openness, communication and relationship satisfaction. The variables of both partners are considered a common dyadic construct, so we use the actor–partner interdependence model (APIM) for the analysis [41] to understand the relationship processes between two people who are in a committed relationship. Taking into account the independence of the dyadic data, as proposed by Kenny [91], the APIM (Figure 1) simultaneously estimates (1) the impact of the wife’s perception of her husband’s openness on her satisfaction (actor effect), and (2) the impact of the husband’s perception of his wife’s openness on her satisfaction (partner effect). In the case of the simple APIM with the perception of a partner’s openness as a predictor and relationship satisfaction as a result, we expect significant actor effects:

**Hypothesis** **1 (H1).** *Women who consider their husbands to be more open experience greater satisfaction. We also expect significant partner effects.*

**Hypothesis** **2 (H2).** *Women who are perceived by their husbands as more open feel greater relationship satisfaction.*

In addition, we propose a model in which the relationship between the perception of a partner’s openness and relationship satisfaction is moderated by communication quality. The analyses were conducted to confirm the following hypotheses:

**Hypothesis** **3 (H3).** *A wife’s high-quality communication weakens the impact of her perception of her husband’s openness on her satisfaction with the relationship.*

**Hypothesis** **4 (H4).** *A husband’s high-quality communication weakens the impact of his wife’s perception of her own openness on relationship satisfaction.*

**Hypothesis** **5 (H5).** *A wife’s high-quality communication weakens the impact of her husband’s perception of his wife’s openness on relationship satisfaction in women.*

**Hypothesis** **6 (H6).** *A husband’s high-quality communication weakens the impact of his perception of his wife’s openness on relationship satisfaction in women.*

Openness is understood as being available, giving love, and willingness to open up to a partner. The study adopted a dyadic model and the following factors were of interest: the importance for relationship satisfaction of women’s own openness, the perceived partner’s openness, and women’s openness as viewed by their partners. Furthermore, we decided to determine how important communication is in perceiving oneself and one’s partner, and the evaluation of communication was treated as a mediator between a wife’s perception of her husband’s openness and her satisfaction with the relationship.

The presented study is part of a larger research project aimed at understanding how the family system works when faced with the loss of a child. The studies included spouses diagnosed with infertility and those who had experienced miscarriage.

## 3. Materials and Methods

### 3.1. Participants

A total of 90 married couples took part in the study (*N* = 180). We examined marriages which had lost a child due to miscarriage or fetal death (50 couples, *N* = 100) and marriages with diagnosed infertility (40 marriages, *N* = 80). The criterion qualifying for the study was the experience of infertility or miscarriage, marital status and consent of both spouses to participate in the study. The average age of spouses who had experienced loss was M = 36.02, SD = 7.45 (M = 35.12, SD = 7.55 for wives, M = 36.92, SD = 7.34 for husbands). For spouses with infertility, the average age was M = 35.82, SD = 7.68 (M = 34.70, SD = 7.88 for wives, M = 36.93, SD = 7.48 for husbands). The average length of marriage for spouses experiencing loss was M = 11.00, SD = 8.07; for those with infertility it was M = 9.23, SD = 6.81. Among those who had lost a child prenatally, 74% had living children as well. In the group of infertile couples, 92% were childless. Those who had lost a child in the prenatal period were asked when the latest pregnancy loss had occurred. In 74% of cases, this was more than a year before. In this group, 82% of spouses had lost a child only once. Others had experienced it two (12%), three (4%), or four or more times (2%). A total of 88% of this group had lost a child due to miscarriage, and 12% as a result of fetal death.

### 3.2. Procedures

The study was conducted within the Opolskie and Śląskie voivodeships in Poland. Access to the examined individuals was possible through gynecologists, midwives, and nurses working in gynecological-obstetric wards. After the candidates for the study gave their consent, they were contacted by phone. Each couple who consented to the study was met individually, most often at their home. The respondents were then given two packages of questionnaires in envelopes, which they could seal after completing. The respondents filled in the questionnaires at their homes, without the presence of the researcher. The researcher then made an appointment to collect the completed questionnaires and talk to the respondents if they expressed such a wish. A total of 103 married couples were examined, but 13 were rejected due to numerous deficiencies in the spouses’ questionnaires. All participants were informed that the study was confidential and that they could withdraw from it at any time. All agreed to participate in the research. All subjects gave their informed consent for inclusion before they participated in the study. The study was conducted in accordance with the Declaration of Helsinki. This piece of research is not by nature a clinical experiment, and as such it did not need to be adjudicated by the Research Ethics Committee.

### 3.3. Measures

The authors created a questionnaire designed specifically for this study that explored partners’ socio-economic backgrounds and the issues of procreation and infertility; it also contained open questions which allowed the participants to express themselves freely in writing (e.g., When was the last pregnancy lost? (a) less than 6 weeks ago; (b) 6 weeks to six months ago; (c) six months to a year ago (d) over a year ago; Has there been a ritual to bury the body of the child/child? (a) yes (b) no (c) in some cases yes, in others, no. If you want to write more about it, please do so now.).

A Polish validation of David Olson’s FACES IV (Flexibility and Cohesion Evaluation Scales) was also used. This validation was created by Andrzej Margasiński, based on the latest, thoroughly revised version of the Circumplex Model [8]. FACES IV is made up of 62 items grouped into eight scales: balanced cohesion and balanced flexibility, disengaged, enmeshed, rigid and chaotic, followed by family communication and family satisfaction [92]. In our analyses, we used the Family Communication Scale, which serves to describe the quality of communication in the family or partner subsystem as perceived by the examined individual [92]. Measured with Cronbach’s alpha, the reliability of this scale is 0.92 [92]. The reliability of the tool, as measured by the value of the Cronbach’s alpha coefficient in our study, was 0.87.

The Marriage Success Scale, developed by Maria Braun-Gałkowska, allowed us to calculate the indicators of marital success. To do this, Braun-Gałkowska understands a respondent’s subjective opinion about his or her relationship satisfaction [93]. The scale is composed of 46 statements. The value of Cronbach’s alpha coefficient in our study was 0.76.

The Giessen Test is used to examine one’s self-image and the image of one’s significant others (spouses, partners, etc.); it comprises 40 bipolar statements to which respondents indicate their level of agreement or disagreement on a continuum ranging from −3 to +3, with 0 being the neutral value [94]. Its items contribute to six scales: Social Resonance, Pliancy, Control, Depressiveness, Openness, and Social Potency [94,95]. For the purposes of this article, only analyses limited to the reservedness–openness dimension are presented. The openness scale characterizes one’s way of relating to and interacting with people. At one end of the scale, an open attitude is manifested, i.e., a sense of security in one’s relations with the world and being open to one’s feelings; at the other end, there are traits showing anxiety and isolation (restraint in expressing feelings and needs, unapproachability to others, distrust, decreased ability to experience love). The value of Cronbach’s alpha coefficient in our study was 0.70.

## 4. Analysis Strategies

The means and standard deviation are calculated for all variables; differences between men and women are examined using the t test for dependent samples. Correlations for each variable between men and women assume non-independence of observations in the dyads [91]. The dependent variable is relationship satisfaction, while the independent variables are each partner’s perception of the other partner’s openness.

Dyadic data were analyzed by the actor–partner interdependence model (APIM) approach [41]. The APIM was developed as a conceptual framework for collecting and analyzing dyadic data, primarily by stressing the importance of considering the interdependence that exists between dyad members. In model 1, which is the base APIM (Figure 1), we examine the relationship between relationship satisfaction and each partner’s perception of the other partner’s openness. In model 2, the effect of both predictors (perception of own openness and perception of partner’s openness) on relationship satisfaction is simultaneously analyzed in order to measure the effect of idealization by controlling for both partners’ perception of their own openness [96]. In this way, we can check whether the relationship between the perception of a partner’s openness and relationship satisfaction exists when we control for the actual characteristics of the partners. As another potential variable that confounds the relationship between relationship satisfaction and the perception of a partner’s openness, the type of stress that the marriage is struggling with (infertility or miscarriage) was added to the analysis in the model 3 (Table 3).

After using APIM to analyze how actor and partner effects can change depending on the characteristics of the spouses and the type of stress experienced in their relationship, we focus on the communication variable, which hypothetically can moderate actor and partner effects of the relationship between the perception of a partner’s openness and relationship satisfaction. We analyze the impact of the interaction between communication and the independent variables (perception of partner’s openness) on relationship satisfaction using the Actor Partner Interdependence Moderation Model APIMoM [97]. The model includes data from both members of the dyads regarding the perception of their partner’s openness as predictors, relationship satisfaction as a result, and communication of both partners as moderators of the actor and partner effects. Potential moderators of effects in APIM include variables whose results differentiate (1) members of a dyad (within-dyad moderator), (2) dyads (between-dyad moderator), or (3) both (mixed moderator). Communication as a moderator is a mixed variable (spouses within one dyad may have different results in this variable; the averaged results of communication of both spouses may also differ between dyads). There are two potential moderators of the actor and partner effects: the actor moderator (own communication) and the partner moderator (partner communication). There are also two actor effects and two partner effects of the moderating variable. Using gender as the differentiating variable, eight interaction effects can be analyzed:

(1) The wife’s communication moderates the relationship between her perception of her partner’s openness and her relationship satisfaction (actor effect)
(2) The husband’s communication moderates the relationship between his wife’s perception of his openness and her relationship satisfaction (partner effect)
(3) The wife’s communication moderates the relationship between her husband’s openness in the husband’s perception and her relationship satisfaction (actor effect)
(4) The husband’s communication moderates the relationship between the husband’s perception of his wife’s openness and his wife’s relationship satisfaction (partner effect)

Similarly, effects 5–7 relate to male satisfaction: (5) male actor effect moderated by a male moderator; (6) male partner effect moderated by a male moderator; (7) male actor effect moderated by a female moderator; (8) male partner effect moderated by a female moderator. Although in ongoing research we focus our attention particularly on women, in the APIM these effects are also calculated.

The hypothesized model was evaluated using goodness-of-fit indices that included the chi-square test, the root mean square error of approximation (RMSEA; acceptable fit ≤ 0.08) [98], and the Comparative Fit Index (CFI; acceptable model fit ≥ 0.9). To create a simpler and more interpretable model, constraints are placed on interaction effects, from which k values can be estimated. k is the ratio of the partner effect to the actor effect. When k is 1, we have a couple-level model; k equal to −1 is a contrast model; k equal to 0 is an actor-only model.

To test the differences between the genders, we calculated the difference between the actor effects of a woman and a man, as well as the difference between the partner effects of a woman and a man. [91]. All tests were performed at the 0.05 significance level. All APIM analyses were performed as part of Structural Equation Modeling SEM; [99] implemented by R’s [100] lavaan package [101], double verified with APIM_SEM [96] and APIMoM apps [97]. All other analyses were performed using the IBM SPSS Statistics (Armonk, NY, USA) 24 statistical package, based on an agreement between the Jagiellonian University and Predictive Solutions (Kraków, Poland), thanks to which employees, students and doctoral students of the Jagiellonian University can use the software included in the PS IMAGO PRO Academic package free of charge until June 30, 2021.

## 5. Results

According to APIMPower app [102], the minimum sample size necessary to detect the actor and partner effects for an Actor-Partner Interdependence Model analysis with distinguishable dyads given a desired level of power (0.80) and alpha (0.05) is 91 dyads. Our sample consists of 90 dyads, so we can conclude that our sample size is relatively small, but still sufficient for the APIM analysis. In the original Giessen Test, the openness scale is based on the results of the opposite end of the scale—reservedness, which must be taken into account when interpreting the results.

The means, standard deviations, and *t* tests for dependent samples, testing the differences between genders and between the groups of infertile spouses and those with an experience of miscarriage, are presented in Table 1. A Levene’s test showed the homogeneity of variance of the compared groups of spouses which had experienced infertility or miscarriage (*p* = 0.169–0.942). Only in the case of communication in men did the test turn out to be statistically significant (*p* = 0.042). The *t* test for independent samples showed significant differences in the level of satisfaction and communication between the studied groups (Table 1). In the later analyses (model 3), we also took into account the type of stress the marriage was struggling with: infertility or miscarriage. We did not observe significant differences between men and women in terms of the values presented. Only men’s perception of their own openness seems to be significantly higher compared to women’s perception of their own openness.

The spouses’ results correlate significantly in terms of relationship satisfaction and communication (Table 2). In men, satisfaction with a relationship is significantly linked to their wife’s communication and perception of their husband’s openness (Spearman rank correlation coefficient (rs) ranged from 0.23 to 0.65). Women’s satisfaction is correlated with their own and their partner’s communication, their perception of their own and their husband’s openness, and their husband’s perception of his own and his wife’s openness (rs ranged from 0.33 to 0.88). The wife’s and husband’s perceptions of their own and their partner’s openness are not correlated between the spouses. On the other hand, the wife’s and husband’s perception of their own openness significantly correlates with the wife’s and husband’s perception of the other partner’s openness in both sexes.

### 5.1. The Spouses’ Perception of Their Partner’s Openness as a Predictor of Marital Satisfaction

A within-dyads variable that distinguishes heterosexual partners from each other across the sample is gender. In order to determine if gender makes a statistically meaningful difference, a model with distinguishable members and a model with indistinguishable members were compared. This overall test of distinguishability was not statistically significant (χ^2^ (6) = 0; *p* = 1.000), therefore we cannot conclude that members can be statistically distinguished based on the gender variable. We are given results of a model that treats the dyad members as indistinguishable. The couple-level pattern is plausible in the presented model (*k* = 1), which indicates that the two actor effects and the two partner effects are set to be equal [43]. Satisfaction of a dyad member is equally influenced by his or her own predictor variable as by the partner’s predictor variable [98].

Before conducting the analysis, the results of independent variables and moderators were centered. Table 3 shows the results of the three models. As expected (H1), the wife’s perception of her husband’s openness significantly (actor effect in women = −0.33; *p* < 0.001) and positively predicts relationship satisfaction in women. The analysis of the partner effect (H2) indicates that a husband’s perception of his wife’s openness also significantly predicts relationship satisfaction in women (partner effect in women = −27; *p* < 0.01). There are no significant differences between the actor effect (*p* = 1.00) and partner effect (*p* = 1.00), which indicates the same pattern in both genders. In contrast, in the context of predictors, the correlation between the satisfaction results for both spouses is equal to 0.52 and is statistically significant (*p* < 0.001). So, if a wife obtains a high/low score on the satisfaction scale due to her positive/negative perception of her husband’s openness or due to her husband’s positive/negative perception of his own openness, then her husband also presents an accordingly high/low score on the satisfaction scale.

The rectangles represent the independent and dependent variables; the two circles present the latent error terms; the arrows describe the actor and partner effects. The curved double-headed arrows on the left represent the covariances between the independent variables; the curved double-headed arrow on the right represents the correlation between the two error terms (e1, e2: residual errors on satisfaction for males and females, respectively). * *p* < 0.05; ** *p* < 0.01; *** *p* < 0.001; standardized coefficients (*β*) are reported with standard error in parentheses.

When we simultaneously, with the wife’s and the husband’s perception of each other’s openness, control for the wife’s and the husband’s perception of their own openness as a measure of the actual characteristics of the spouses (model 2), we observe that women who present a more idealized image of their partner are more satisfied with the relationship (actor effect = −25, *p* < 0.001). Similarly, women who are more idealized by their partners present higher satisfaction (partner effect = −0.21, *p* < 0.001). It seems that the more women idealize their husbands, the more they are satisfied with the relationship. When we test the impact of the spouses’ perception of their own openness on satisfaction, both the actor effect (−0.13; *p* = 0.009) and the partner effect (−0.18; *p* < 0.001) prove to be statistically significant, which indicates that the more the partners assess themselves and each other as open, the more satisfied they are with the relationship.

Model 3 contains two potential confounding variables: (1) the wife’s and the husband’s perception of their own openness, and (2) the type of stress experienced by the spouses, i.e., infertility or miscarriage. The analysis shows that despite controlling for these variables, the actor effect (−0.24; *p* < 0.001) and partner effect (−0.20; *p* < 0.001) still remain statistically significant. We conclude that in the context of the analyzed potential confounding variables, the relationship between the wife’s and the husband’s perception of each other’s openness and relationship satisfaction in women is strong. In addition, we observed that relationship satisfaction in women from the group of infertile marriages is 6.06 points lower compared to women from the group of marriages after miscarriages (*p* = 0.034).

### 5.2. Communication as a Moderator of the Impact of the Wife’s and the Husband’s Perception of Each Other’s Openness on Relationship Satisfaction

The dyadic members are distinguishable based on the basis of gender, but the effects of the independent variable, the moderator, and their interactions are the same for both partners, and the test of distinguishability is not statistically significant (χ^2^ (8) = 0.00, *p* = 1000, RMSEA = 0.000). Given the value of the RMSEA and the fact that the chi-square test is not statistically significant, the interaction effects should be treated as equal for all members; however, it should be remembered that even if the moderator effects are indistinguishable, the actor and partner effects may still be distinguishable [102].

**Figure 2:**
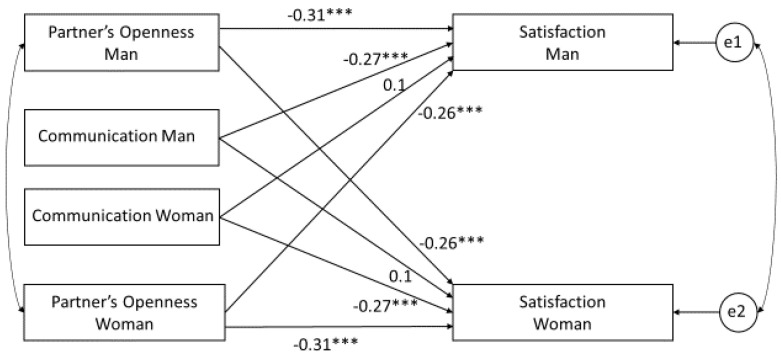
Generic actor–partner interdependence model with communication as the moderator. The rectangles represent the independent and dependent variables; the two circles (e1, e2) present the latent error terms; the single-headed arrows show regression coefficients (the actor and partner effects); the curved double-headed arrow on the right represents the correlation between the two error terms. Although not shown in the model, the communication–openness interactions were included as covariates. All predictors, including interaction terms, are allowed to covary. Note: “openness” is the opposite pole of “reservedness”. * *p* < 0.05; ** *p* < 0.01; *** *p* < 0.001.

Before conducting the analysis, the results of the independent variables and moderators were centered. The effects of two models were compared: the model without interaction (Figure 1) and the moderation model (Figure 2). The model without interaction does not present good fit indicators (χ^2^ (4) = 58.87, *p* < 0.001, RMSEA = 0.276). In the moderation model (Table 4), we observe (1) statistically significant actor and partner effects of the independent variable X (the husband’s and wife’s perception of each other’s openness) on the dependent variable Y (relationship satisfaction); (2) the significant actor effect of the moderator (communication) on the dependent variable Y (satisfaction (the partner effect turned out to be statistically insignificant)); (3) the statistically significant actor–actor effect of the interaction between each partner’s perception of the other partner’s openness and communication on satisfaction; (4) the statistically significant actor–partner effects of the interaction between the perception of a partner’s openness and communication on satisfaction. The partner–actor effect (the impact on the wife’s satisfaction of the interaction between each partner’s perception of the other partner’s openness and the husband’s communication) and the partner–partner effect (the impact on the husband’s satisfaction of the interaction between each partner’s perception of the other partner’s openness and the husband’s communication) were not statistically significant.

Confidence intervals for the *k* value were created using the Monte Carlo method—the parametric bootstrap. Tests of specific dyadic patterns [103] confirmed the results above: the *k* value for the independent variable is 0.126 (CI: −4.273 to −1.216), which indicates the contrasted X pattern; the *k* value for the moderator is −1.867 (CI: −0.133 to 0.921), which indicates the actor M only pattern. Only the actor moderator is used in the actor M only pattern. The contrast X pattern means that the moderation of the actor effect by the actor and partner moderator variables is equal but in the opposite direction [96], i.e., both the actor effect and the partner effect weaken as the actor’s moderation variable increases. In other words, the greater the communication quality, the smaller the impact on relationship satisfaction of each partner’s perception of the other partner’s openness. The test of the constrained interaction model indicates a good fit of the model to the data (χ^2^ (1) = 2.34, *p* = 0.126, RMSEA = 0.086), which confirms that the presented model explains the interaction effects formula.

## 6. Discussion

The purpose of the presented analysis was to determine the impact of openness and communication on the relational satisfaction of women facing infertility or a miscarriage. Communication was considered a moderator of the relationship between relationship satisfaction and the wife’s and the husband’s perception of their own and their partner’s openness. The dyadic approach was adopted, and the actor–partner interdependence (APIM) was used in the analyses.

Although the *t* test for independent samples showed significant differences in the level of spouses’ communication and satisfaction between the studied groups, in the dyadic level of analyses only one difference was observed between women who had had a miscarriage and those who were infertile, and it concerned the level of relationship satisfaction. Women who had experienced miscarriage presented a higher level of satisfaction than those who had experienced infertility. This difference could be explained by the fact that infertility, which is a source of tremendous stress, is a more long-term condition, and chronic stress can have a greater impact on relationships between partners than stress caused by miscarriage, which is rather acute but short-lived [6]. Research indicates that the longer the struggle with infertility and treatment that does not bring the desired results, the lower the marital satisfaction [44,104,105]. Infertility can also result in lower hopes of having a child and thus adversely affect the experiences and emotions of partners and the mutual relations between them, as indicated by the literature. It is also worth paying attention to the fact that infertile couples and those after miscarriage are perceived differently by society. Sometimes the former is perceived less positively and—due to the lack of children—they are considered selfish and only interested in focusing on a comfortable and prosperous life. Social pressure and the negative perception of childless marriages can be a source of additional stress that affects relationship satisfaction.

Our results show that the wife’s perception of her husband’s openness significantly and positively predicts relationship satisfaction in women. Additionally, the husband’s assessment of his wife’s openness turns out to be associated with women’s relationship satisfaction. The results obtained are consistent with the results of other studies that indicated the importance for the quality of relationships of openness between partners [4,17]. Openness is valued by partners [18], therefore it is understandable that if partners see themselves and each other as open, this can be conducive to building good and satisfying relationships. In the context of the partner’s self-perceived openness, each spouse’s level of marital satisfaction is associated with their partner’s satisfaction. Openness to a similar extent determines the spouses’ relationship satisfaction. This means that the spouses are congruent in their own assessment of each other’s openness, which is related significantly to their relationship satisfaction. Congruence is indicative of satisfaction [106]. A high congruence of perception in marriages indicates a more appropriate response to the partner, more accurate expectations, and a better anticipation of the other’s feelings. As a consequence such a marriage will reflect a higher degree of satisfaction for both spouses.

Our research results show a strong relationship between each spouse’s perception of their partner’s openness and their satisfaction with the relationship; this is in line with other studies indicating that each partner’s image of the other partner is an important predictor of relationship functioning [83]. Interestingly, the results obtained lead to the conclusion that the more spouses idealize their partners and are idealized by them, the more the both are satisfied with the relationship. This is in line with research that reports that the more positively the partner is perceived, the higher the satisfaction with the relationship [107]. It is also understandable that if spouses value openness, the higher the spouses rate their partners in this respect, the more positive they are towards them and the more satisfied they are with their relationship.

The analyses made it possible to determine the importance of marital communication as a moderator of the relationship between partners’ openness and relationship satisfaction in women. The analyses show that the more communication is valued by spouses, the smaller the impact on relational satisfaction of each partner’s perception of the other partner’s openness. This allows us to argue for the importance of the quality of partner communication for women’s satisfaction with marriage. We can also presume that if a wife assesses communication with her partner as good, then it is less important how she assesses his mutual openness. Good-quality communication seems to guarantee marital satisfaction even when each partner’s perception of their partner’s openness is less positive. As was indicated in other studies, the obtained results confirm the importance of communication for the quality of relationships and relationship satisfaction [4,108]. Considering that we studied couples experiencing stress resulting from infertility or miscarriage, the result which indicates the importance of communication for relational satisfaction may indirectly confirm the thesis that communication is an important element of dyadic coping with stress [6]. This result is also consistent with research results that indicate that partners want to feel that they can talk openly on sensitive topics [15,16], and open communication promotes relational satisfaction [2]. The high-quality communication rating, which translates into satisfaction, allows us to conclude that open communication helps partners to survive a difficult period, as was noted in a study of spouses who had lost their child as a result of miscarriage [40,75]. Sharing difficult experiences, talking about losing a child, or not having a child can increase marital satisfaction. This conclusion is so important that, as clinical experience shows, spouses often avoid talking about infertility or losing a child as a result of a miscarriage because these subjects are taboo. In the case of infertile spouses, the lack of discussion on this subject may be part of their strategy of avoiding the problem in order to avoid stigmatization.

The obtained results also indicate a relationship between openness and communication, thus allowing us to conclude that when partners have a more positive opinion of each other’s openness, the quality of communication is higher. Therefore, they confirm that openness is an important element of communication between partners that defines its quality [4]; the results also allow us to conclude that openness is an important characteristic of romantic relationships (e.g., [80,81,82]), which makes spouses evaluate their relationships and communicate better. Openness facilitates the sharing of emotions and experiences, and it increases the willingness to listen and support a spouse, which in the case of married couples struggling with a difficult experience such as the loss of a child seems to be of particular importance.

## 7. Conclusions

The conducted research indicates a link between relationship satisfaction and the communication and openness of partners. Women’s satisfaction is associated with their own communication, their husband’s self-perceived openness and communication, and his perception of his wife’s openness. Both spouses’ perception of their own openness is related to their perception of their partner’s openness. In turn, each partner’s perception of the other partner’s openness significantly predicts the wife’s satisfaction with the relationship. The results obtained show the same pattern in both sexes. The conducted analyses allow us to conclude that if a woman obtains a high/low result on the satisfaction scale due to a positive/negative perception of her partner’s openness (as assessed by herself and her husband), then the man also presents a high/low score on the satisfaction scale. There are grounds to believe that the more a woman idealizes her husband, the more she is satisfied with the relationship. Additionally, women who are more idealized by their partners present a higher satisfaction. It turns out that the better the partners assess themselves and each other as open, the more satisfied they are with the relationship.

The conducted analyses lead to the conclusion that there is a strong relationship between the perceived image of a partner’s openness and women’s satisfaction with the relationship. In addition, we observe that relationship satisfaction in women from the group of infertile marriages is lower compared to women from the group of marriages after a miscarriage. We also found that the greater the communication quality, the lower the impact on relationship satisfaction of each partner’s perception of the other partner’s openness.

The research undoubtedly brings us closer to understanding the determinants of women’s satisfaction with relationships in general, especially in the case of experiencing difficult and traumatic situations, which undoubtedly includes infertility or the loss of a child in the prenatal period. Importantly, the analyses made it possible to determine the significance of not only specific characteristics of women and their partners (openness, quality of communication), but also the importance of mutual assessment of partners’ openness (husband’s image in the eyes of the wife and wife’s image in the eyes of the husband) for the satisfaction they derive from being in close relationship.

An undoubted advantage of the research is its dyadic character and ability to determine the importance for perceived relationship satisfaction of both partners’ assessment of their own and each other’s openness and communication. In terms of relationship satisfaction, this research shows the interdependence of results in the dyad, the impact of spouses’ openness in both partners’ perception, as well as a moderating role of communication. It is worth noting that dyadic analyses that allow comparisons to be made between the actor and partner effects are not very common in studies of infertile couples and those who have experienced miscarriage [109].

### Limitations

The limitation of this research is the relatively small number of respondents. Analyses using the APIMPower application [102] confirmed that our sample size is sufficient for APIM analysis, however it is still relatively small and all generalized conclusions must be treated with caution. A relatively small group of respondents made it impossible to perform separate APIM analyses for both compared groups. It would be especially desirable if there were significant differences in the communication and satisfaction between them. In future studies, it would also be worth examining couples who do not have problems with having children in order to be able to check to what extent the situation of those experiencing loss is specific in terms of the factors that are relevant to perceived relationship satisfaction and the importance of openness for the quality of relationships. It would also be worth looking into the relationship between communication and openness in future research. Assuming that general communication competence consists of many more elementary competences (ability to listen, mindfulness, empathy, correct reading of verbal and nonverbal messages, understanding the partner, etc.), it would be interesting to determine how specific communication components translate into openness and, as a consequence, into satisfaction, and which one has the greatest impact on relational satisfaction.

## Figures and Tables

**Table 1:** Descriptive statistics, the group (infertility and miscarriage) and the gender differences tests.

	Men		Women		Men & Women		*t* Test for Group Differences (Men/Women)	*t* Test for Gender Differences
	M	SD	M	SD	M	SD		
Satisfaction	38.53	7.40	37.33	8.51	37.93	7.98	2.75 **/3.87 ***	−1.93
Communication	23.38	4.67	37.63	8.33	30.51	9.82	2.55 */3.47 **	−1.57
Reservedness_S	21.21	4.74	21.13	5.61	21.17	5.18	−1.69/−0.86	−3.01 **
Reservedness_P	38.94	6.46	22.84	6.30	30.89	10.28	−1.00/−1.22	1.91

* *p* < 0.05; ** *p* < 0.01; *** *p* < 0.001. Openness_O: perception of own openness; Openness_P: perceived partner’s openness. Note: “openness” is the opposite pole of “reservedness”.

**Table 2:** Pearson’s correlations between satisfaction, communication and openness.

		1	2	3	4	5	6	7	
1	Satisfaction_A	−							
2	Communication_A	0.88 **	−						
3	Openness_O_A	−0.25 *	−0.20	−					
4	Openness_P_A	−0.35 **	−0.40 **	0.02	-				
5	Satisfaction_P	0.65 **	0.62 **	−0.15	−0.23 *	−			
6	Communication_P	0.59 **	0.59 **	−0.23 *	−0.18	0.86 **	-		
7	Openness_O_P	−0.22 *	−0.27 *	0.07	0.40 **	−0.10	−0.13	−	
8	Openness_P_P	−0.33 **	−0.32 **	0.29 **	−0.05	−0.27 **	−0.23 *	0.14	−

* *p* < 0.05; ** *p* < 0.01 (*n* = 90 dyads). _A: women ratings; _P: men ratings; Openness_O_A/P: perception of own openness in women/men; Openness _O_A/P: perceived partner’s openness s in women/men. Note: “openness” is the opposite pole of “reservedness”.

**Table 3:** Actor and partner effects of perceived partner’s openness on marital satisfaction.

	Effect	Estimate	95% CI	*p*	Beta	*R*
Model 1	Intercept	69.53	66.90 to 72.16	<0.001		
	Actor	−1.31	−1.70 to −0.92	<0.001	−0.33	−0.33
	Partner	−1.08	−1.47 to −0.70	<0.001	−0.27	−0.28
	*K*	0.83	0.61 to 1.02			
Model 2	Openness_P					
	Intercept	69.53	66.99 to 72.07	<0.001		
	Actor	−1.01	−1.42 to −0.60	<0.001	−0.25	−0.26
	Partner	−0.85	−1.26 to −0.44	<0.001	−0.21	−0.21
	*K*	0.84	0.55 to 1.11			
	Openness_O					
	Intercept	69.53	66.99 to 72.07	<0.001		
	Actor	−0.57	−0.10 to −0.14	0.009	−0.13	−0.13
	Partner	−0.80	−1.23 to −0.37	<0.001	−0.18	−0.15
	*K*	1.406	0.89 to 2.26			
Model 3	Openness_P					
	Intercept	72.22	68.83 to 75.62	<0.001		
	Actor	−0.96	−1.37 to −0.56	<0.001	−0.24	−0.25
	Partner	−0.80	−1.21 to −0.39	<0.001	−0.20	−0.21
	*k*	0.83	0.52 to 1.11			
	Openness_O					
	Intercept	72.22	68.83 to 75.62	<0.001		
	Actor	−0.52	−0.95 to −0.10	0.016	−0.123	−0.13
	Partner	−0.75	−1.18 to −0.33	<0.001	−0.18	−0.14
	*k*	1.44	0.87 to 2.45			

Openness_O: perception of own openness; Openness_P: perceived partner’s openness. Note: “openness” is the opposite pole of “reservedness”.

**Table 4:** Effects in the moderation model.

Cause	Type	Estimate	95% CI	*p*	Standardized
X	Actor	−0.89	−1.28 to −0.49	< 0.001	−0.31
	Partner	−0.75	−0.22 to −0.35	< 0.001	−0.26
M	Actor	−1.08	−1.47 to −0.70	< 0.001	−0.27
	Partner	0.05	−0.22 to 0.33	0.696	0.01
Interaction	Actor-Actor	0.08	0.02 to 0.13	0.003	0.16
	Actor-Partner	−0.13	−0.18 to −0.07	< 0.001	−0.25
	Partner-Actor	0.05	−0.01 to 0.11	0.082	0.10
	Partner-Partner	−0.02	−0.08 to 0.030	0.395	−0.05

Note: “openness” is the opposite pole of “reservedness”.
